# Expanding the Spectrum: A Rare Case of Morquio Syndrome With Bronchial Asthma and Seizure Disorder

**DOI:** 10.1002/ccr3.71123

**Published:** 2025-10-06

**Authors:** Bijay Bastola, Sweekar Dahal, Ramesh Raj Acharya, Manish Sah, Pratisma Wagle

**Affiliations:** ^1^ Manipal College of Medical Sciences Pokhara Nepal; ^2^ Lalbandi Municipality Hospital Sarlahi Nepal; ^3^ Department of Pediatrics Manipal College of Medical Sciences Pokhara Nepal

**Keywords:** bronchial asthma, case report, Morquio syndrome, multisystem involvement, seizure disorder

## Abstract

This case highlights a rare co‐existence of Morquio syndrome with bronchial asthma and myoclonic seizures, expanding the known clinical spectrum. It emphasizes the need for comprehensive, multidisciplinary management to address multisystem involvement and describes the challenges of treatment in resource‐limited settings where advanced therapies and diagnostics may not be readily available.

## 1 Introduction

Mucopolysaccharidosis (MPS) type IV, or Morquio syndrome, is a rare, autosomal recessive lysosomal storage disease. Mucopolysaccharidoses, in general, are a group of inherited metabolic disorders characterized by deficiencies in specific lysosomal enzymes that are required for the breakdown of mucopolysaccharides. These enzyme deficiencies lead to the accumulation of mucopolysaccharides in various tissues and organs, resulting in the clinical disease [1, 2]. In Morquio syndrome, the accumulation is of glycosaminoglycans or GAGs, such as chondroitin‐6‐sulfate and keratan sulfate [3].

Two types of Morquio syndrome have been described: Type A and Type B. Type A is due to a mutation in the galactosamine‐6‐sulfatase enzyme in the GALNS gene, while Type B, usually milder than Type A, is due to a mutation in the Beta‐galactosidase enzyme in the GLB1 gene [2, 3]. The incidence of Morquio syndrome ranges from 1 in 71,000 in the United Arab Emirates to 1 in 500,000 in Japan [4].

Chondroitin‐6‐sulfate and keratan sulfate are primarily produced in cartilage [5]. Thus, disruption of their metabolism leads to defective osteogenesis and chondrogenesis, which are the major presenting manifestations of Morquio syndrome. It mainly presents as short stature, vertebral or long bone deformities [6].

Traditionally, Morquio syndrome was not believed to be associated with any neurological conditions, but some degree of behavioral and neurological problems like anxiety/depression and somatic complaints [7] has been highlighted in the literature recently. Nonetheless, there are scarce reports of its association with a seizure disorder.

Respiratory complications such as obstructive sleep apnea, tracheal stenosis secondary to an anomalous brachiocephalic artery, and thoracic cage deformities resulting in restrictive lung disease are well‐established manifestations of Morquio syndrome [8, 9]. Although asthma has not been specifically documented in association with Morquio syndrome, several reports have described the coexistence of mucopolysaccharidoses (MPS) with asthma or asthma‐like symptoms [10]. These observations suggest that airway hyperresponsiveness or bronchial asthma may not be merely coincidental, but rather an underrecognized feature within the broader phenotypic spectrum of MPS.

The co‐existence of Morquio syndrome with seizure disorder and bronchial asthma, and challenges in the crucial management of the disease is the unique aspects of our case.

## 2 Case Report

### 2.1 History and Evaluation

A 6‐year‐old male had presented to our tertiary care center with a history of recurrent chest infections since the age of 3 years, with complaints of fever, cough, and rapid breathing during each episode. During his hospital stay at that time, a diagnosis of bronchial asthma was made, and he was discharged on inhalational therapy. Despite regular adherence to treatment, dosage adjustments, and the addition of new medications when necessary, he continued to experience annual asthma exacerbations.

At the age of 9 years, his parents observed reduced growth and bulkiness of his body. They also noted that his body morphology closely resembled that of his sister, who had a similar illness characterized by musculoskeletal dysplasia and had passed away at the age of 18. When asked about the family history, a history of consanguinity was denied. In light of these concerns, regular follow‐up was recommended for growth monitoring and further evaluation.

At the age of 14, he presented with his first episode of generalized abnormal body movement with post‐ictal confusion. During his hospitalization, he experienced multiple similar episodes. Electroencephalography (EEG) confirmed a diagnosis of myoclonic seizures. Magnetic Resonance Imaging (MRI) with seizure protocol revealed no significant abnormalities, except for a few small T2/FLAIR hyperintense foci in the periventricular white matter of the bilateral frontal and parietal lobes, attributed to post‐ictal changes. He was started on Sodium Valproate 200 mg per day. However, due to inadequate seizure control, the dosage was gradually increased during follow‐up visits. Eventually, a second anti‐epileptic medication, Levetiracetam, was added. Currently, he is on sodium valproate 1000 mg per day and levetiracetam 1000 mg per day, with satisfactory seizure control.

Due to his history of recurrent asthma exacerbations, reduced growth and body bulkiness, and multiple seizure episodes, comprehensive investigations and multidisciplinary consultations were performed.

A simultaneous neurological and psychiatric evaluation was conducted. His parents stated that his formal education was limited to the completion of middle school. He appeared agitated during the time of examination. Speech, memory, sensory, motor, and cranial nerve examinations were found to be normal. His thought, attention, judgment, and concentration were appropriate. Intelligence was tested via the Wechsler Adult Intelligence Scale. He scored 81 on the scale, which was categorized as below‐average intelligence. Insight into his health and physical well‐being was present. No definite psychiatric comorbidity was identified.

Otorhinolaryngological evaluation revealed severe mixed hearing loss in the right ear and moderate mixed hearing loss in the left ear. Tympanometry demonstrated a Type B curve in the right ear and a Type C curve in the left ear. Findings suggestive of allergic rhinitis were also noted.

Ophthalmological examination showed a visual acuity of +0.5/−1.5 diopters at 20 degrees (−6/36) in the right eye and +0.5/−1.25 diopters at 160 degrees (−6/36) in the left eye. Additional findings included developmental cataracts, bilateral optic disc pallor, normal corneas, pseudo‐proptosis, and nystagmus.

His most recent echocardiography revealed myxomatous changes in the anterior and posterior leaflets of the mitral valve, with anterior leaflet prolapse and moderate mitral regurgitation. Myxomatous changes were also noted in the aortic and the tricuspid valve, associated with moderate aortic and mild tricuspid regurgitation, respectively. Mild pulmonary arterial hypertension (PAH) was noted, with a pulmonary artery pressure of 35 mmHg. His left ventricular ejection fraction (LVEF) was 65%.

His most recent spirometry evaluation demonstrated FEV1 of 30% and FEV1/FVC of 73%. Post‐bronchodilator spirometry showed a 13% rise from baseline FEV1, indicative of Bronchial Asthma.

**FIGURE 1:**
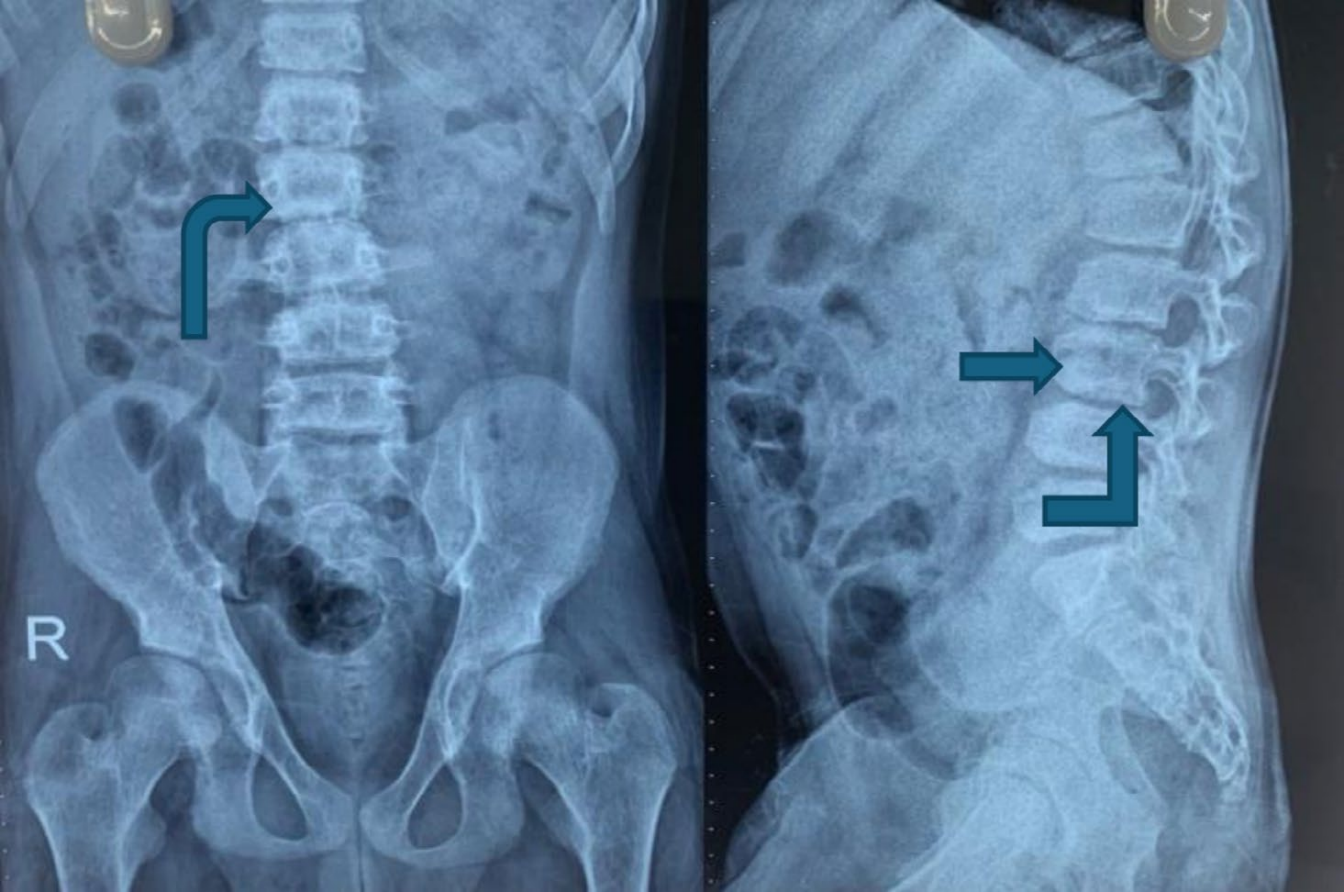
Slight scoliosis with anterior wedging of vertebrae.

**FIGURE 2:**
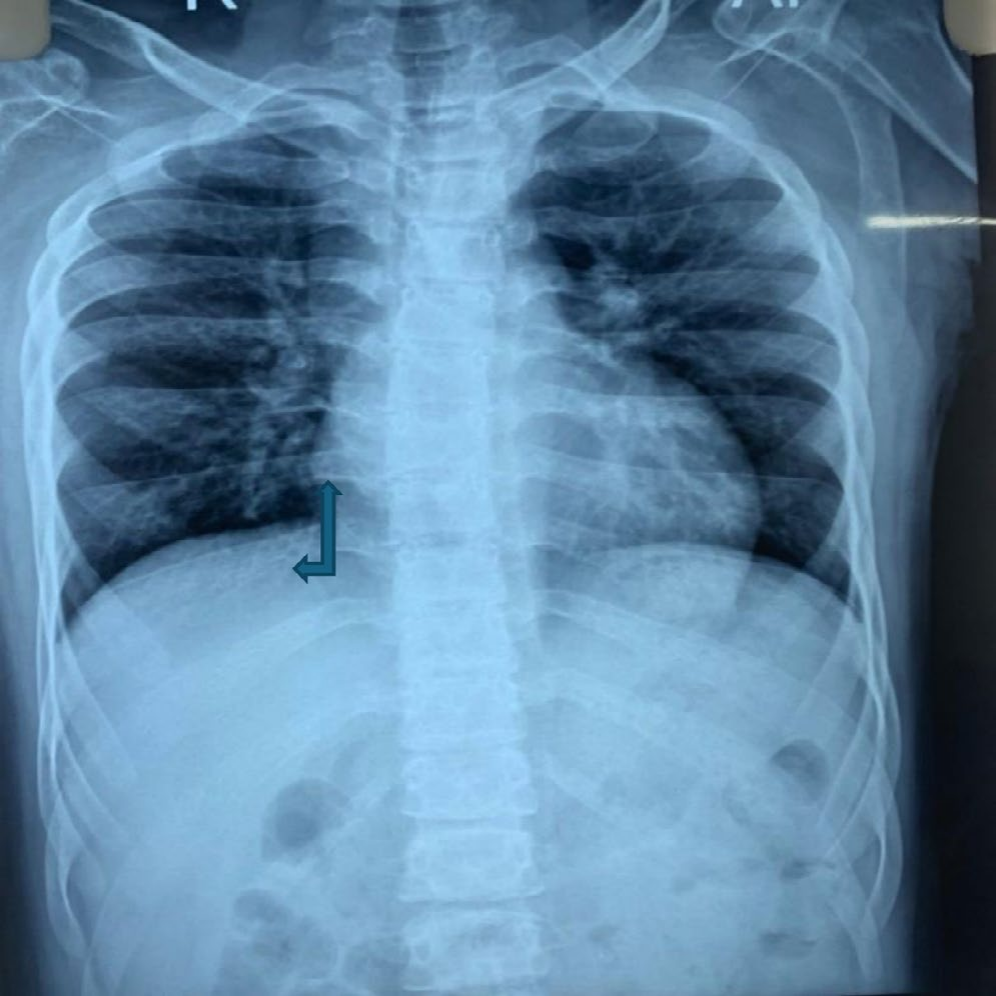
Bilateral perihilar infiltrate suggestive of LRTI with paddle‐shaped ribs.

**FIGURE 3:**
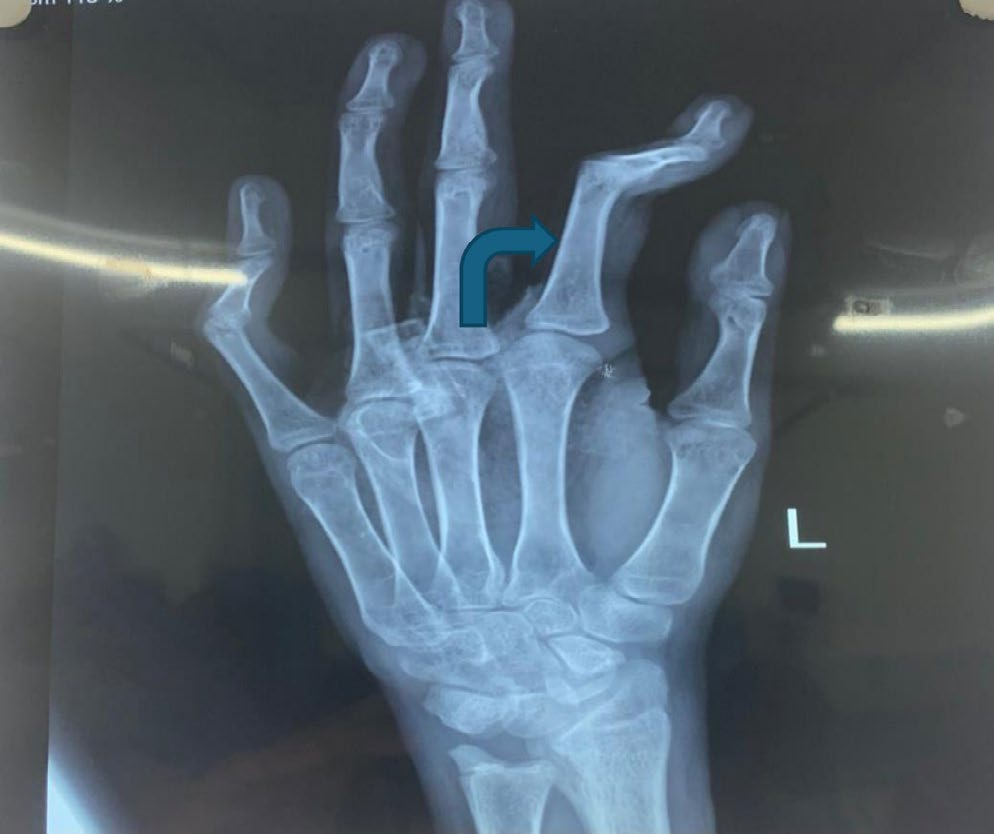
Bullet shaped proximal phalanx.

A skull radiograph demonstrated widened dental spacing and a thickened diploic space. Thoracolumbar spine radiographs revealed anterior vertebral body wedging (Figure 1), platyspondyly, and increased interpedicular distance. Pelvic imaging showed an enlarged, oval‐shaped pelvic inlet accompanied by flared, rounded iliac wings. Chest radiography identified paddle‐shaped ribs (Figure 2). Bullet‐shaped proximal phalanx was also noted (Figure 3). Findings from the comprehensive skeletal survey were consistent with dysostosis multiplex.

Urinalysis was positive for elevated levels of keratan sulfate.

His most recent visit to our hospital was at the age of 20, presenting with an acute onset of shortness of breath and bilateral diffuse wheeze. He was managed in the line of acute exacerbation of asthma and showed improvement with inhaled plus injectable corticosteroid therapy in the emergency.

### 2.2 Diagnosis

Given the strong clinical suspicion, in conjunction with radiological, ophthalmological, and audiological findings, as well as the presence of keratan sulfate in the urine, a diagnosis of Morquio syndrome (MPS IVA) was established.

### 2.3 Outcome

Following his management in the emergency room during his last visit, his maintenance therapy was escalated to moderate‐intensity inhaled corticosteroid with montelukast in a dose of 800 mg and 10 mg per day, respectively. Inhaled salbutamol (2 puffs) was prescribed for acute attacks as a reliever medication. Sodium valproate 1000 mg per day and levetiracetam 1000 mg per day were continued for his seizures. He is being managed with an ACE inhibitor and a beta‐blocker (metoprolol), weighing the risk–benefit ratio for asymptomatic heart failure and potential exacerbation of his asthma. He remains under multi‐disciplinary care and is on regular follow‐up.

Currently, enzyme replacement therapy and hematopoietic stem cell transplant represent the gold standard in the management of Morquio syndrome. However, limited availability and high cost are major barriers to accessing these advanced therapies in this case. Therefore, in resource‐limited settings like ours, conventional treatment modalities remain the only viable option for managing such a complex disease and improving the patient's quality of life.

## 3 Discussion

Morquio syndrome, or Mucopolysaccharidosis IVA (MPS IVA), is a rare autosomal recessive lysosomal storage disorder caused by a mutation in the galactosamine‐6‐sulfatase (*GALNS*) gene in type A, and beta‐galactosidase deficiency in type B disease. The enzymatic deficiency impairs the degradation of glycosaminoglycans (GAGs), particularly keratan sulfate and chondroitin‐6‐sulfate, resulting in their progressive accumulation in various tissues and leading to multisystem involvement [1].

MPS IVA is frequently associated with multisystem complications, especially affecting the cardiopulmonary system. Respiratory manifestations are well documented and include obstructive sleep apnea, tracheal stenosis, which are often related to anatomical anomalies such as an aberrant brachiocephalic artery, and restrictive lung disease [8]. While traditionally classified as metabolic disorders, mucopolysaccharidoses (MPS) are being increasingly recognized for their immunological involvement. Innate immune abnormalities have been well documented across MPS subtypes, but emerging evidence suggests that the adaptive immune system may also play a critical role [11]. Notably, neurological manifestations in MPS III (Sanfilippo syndrome) have been linked to autoimmune‐mediated neuroinflammation. In a murine model, cytotoxic T cells isolated from MPS III mice were shown to induce inflammatory changes and neuromotor symptoms when transferred to wild‐type mice, indicating a potential autoimmune mechanism contributing to neurological decline [11].

In addition to central nervous system involvement, airway‐related symptoms such as macroglossia, enlarged tonsils, reactive airway disease or asthma, and sleep disturbances have been frequently observed in MPS I. Pamela Arn et al. reported these symptoms in 85% of Hurler, 83% of Hurler‐Scheie, and 65% of Scheie syndrome patients, often preceding the diagnosis [10]. Although true hypersensitivity syndromes in MPS are considered rare, isolated reports, including bronchial asthma in MPS VI, have been documented [12]. Although no cases have directly linked bronchial asthma to Morquio syndrome (MPS IV) to date, the known association of MPS with autoimmunity and hypersensitivity suggests that such symptoms may be more than coincidental. Instead, they may represent immunological manifestations inherent to specific MPS subtypes.

Our observations further support the growing hypothesis that immune dysregulation may constitute a fundamental component of the MPS disease spectrum. This warrants further investigation to better elucidate the immunopathological mechanisms involved, which could significantly improve early diagnosis, monitoring, and targeted therapeutic strategies.

Unlike MPS I and II, MPS IV is not associated with progressive neurocognitive deterioration. Most patients maintain normal cognitive function. Nevertheless, impairments in behavior, attention, and executive function have often been overlooked, potentially impacting patients' quality of life. Recent research suggests that these neuropsychiatric manifestations may result from a complex interplay of lysosomal dysfunction, mitochondrial impairment, and altered calcium homeostasis [7]. Supporting this, our patient experienced recurrent seizure episodes over several years, which were unresponsive to initial low‐dose antiepileptic therapy, indicating central nervous system involvement.

Hearing loss is another commonly reported feature in MPS IVA. A systematic review and meta‐analysis by Diaz‐Ordoñez et al. identified hearing impairment in 210 of 568 patients, with conductive hearing loss being the most frequent type and moderate hearing loss the most common in terms of severity. Approximately 19.2% of patients had a history of recurrent otitis media, likely attributable to glycosaminoglycan (GAG) accumulation within the tympanic membrane and ossicular chain [13].

On the other hand, ophthalmological manifestations in Morquio syndrome are generally less severe compared with other systemic features. The most commonly observed ocular abnormality is mild to moderate corneal clouding [14]. Additionally, high myopia and astigmatism are frequently reported. Less commonly, optic atrophy, congenital cataracts, retinal degeneration, and glaucoma have also been documented [14, 15, 16]. The audiological and ophthalmological findings in our patient broadly align with previously described patterns.

In most cases, a probable diagnosis of Morquio syndrome is established based on a strong clinical suspicion supported by radiological evidence. Radiological features typically include spinal deformities such as scoliosis, kyphosis, gibbus deformity, and platyspondyly, along with odontoid hypoplasia and widened intervertebral disc spaces. Pelvic findings often show acetabular narrowing, widening of the pubic symphysis, and iliac flaring. Hand abnormalities may involve shortened metacarpals, small or absent carpal bones, and radial‐ulnar inclination, indicating a complex skeletal dysplasia [17]. Additional specificity in diagnosis can be achieved by demonstrating elevated levels of keratan sulfate in blood or urine [18]. More recently, assessment of N‐acetylgalactosamine‐6‐sulfatase (GALNS) enzyme activity has been recognized as a more specific diagnostic tool [18]. However, due to its limited availability and high cost, clinical evaluation, radiological evidence, and, most importantly, urinary keratan sulfate levels remain the primary means of diagnosis in many settings.

Currently, management strategies for Morquio syndrome type IV include enzyme replacement therapy (ERT) with elosulfase alfa and hematopoietic stem cell transplantation (HSCT) [19, 20]. Clinical trials of weekly elosulfase alfa administration have demonstrated improvements in physical endurance, as measured by the six‐minute walk test, three‐minute stair climb test, and respiratory function assessments, compared to placebo [20]. However, both ERT and HSCT have shown limited efficacy in correcting skeletal abnormalities, primarily due to inadequate delivery of therapeutic agents to avascular cartilaginous lesions [21].

In contrast to earlier findings, a recent study by Yalchin et al. [22] reported encouraging short‐term outcomes in a cohort of 10 pediatric patients (3 females and 7 males, aged 35 to 186 months) who underwent allogeneic HSCT. The study observed increased linear growth and improved functional capacity in daily activities. Although promising, these results require confirmation through larger, multicenter studies with long‐term follow‐up to assess their durability and safety.

Emerging bone‐targeted therapeutic strategies, such as gene therapy using viral vectors, have shown promising results in preclinical models and may offer a future avenue for addressing the skeletal manifestations of the disease [23, 24]. While the efficacy of ERT in managing hearing loss remains uncertain, isolated case reports have documented partial improvement [25].

In this case, the management of MPS IVA becomes considerably more challenging due to the presence of unreported and under‐reported comorbidities such as bronchial asthma and seizure disorder, which is further complicated by asymptomatic heart failure. Our individualized therapy incorporated a cardio‐selective beta‐blocker and an angiotensin‐converting enzyme (ACE) inhibitor, which resulted in effective cardiac optimization, as demonstrated by a stable ejection fraction over a 4‐year follow‐up period. Asthma control was concurrently achieved through the use of inhaled corticosteroids and montelukast. The potential role of glycosaminoglycan (GAG) accumulation in the airways as a contributing factor to persistent asthma symptoms further complicated the clinical picture.

A significant limitation in the management of this patient was the unavailability of enzyme replacement therapy (ERT), a constraint commonly encountered in resource‐limited settings, which may have otherwise substantially improved the patient's quality of life. Nonetheless, through consistent counseling and supportive interventions aimed at enhancing sensory stimulation and comfort, the patient was able to successfully reintegrate into school and improve overall psychosocial functioning. A multidisciplinary care model, along with referral to specialized centers offering comprehensive rehabilitation, has contributed to a significant increase in life expectancy for individuals with Morquio syndrome from an average of 17.42 ± 9.54 years to 30.75 ± 10.84 years [26].

This case emphasizes the need to recognize atypical or under‐reported manifestations of Morquio syndrome, such as bronchial asthma and seizure disorder, which may be part of a broader and more complex disease spectrum. The rare co‐occurrence of severe asthma and mucopolysaccharidoses, with frequent exacerbations and only partial response to conventional therapies, poses a significant clinical challenge. This highlights the necessity for targeted therapeutic strategies.

Eotaxin, a potent chemoattractant for eosinophils that binds to the CCR3 receptor, has been increasingly studied for its role in airway hyperresponsiveness and asthma [27]. CCR3‐mediated eosinophilic migration is now recognized as a key contributor to asthma pathophysiology. Promising novel therapies include compounds like Astragalin, which inhibits endotoxin‐induced oxidative stress, eosinophilia, and epithelial apoptosis in the airways [27]. Additionally, a novel peptide nanoparticle CCR3 inhibitor, compound R321, has shown potent antagonistic effects on CCR3 signaling. In vivo studies demonstrate that R321 effectively blocks eosinophil recruitment to the blood, lungs, and airways, and prevents airway hyperresponsiveness in eosinophilic asthma models [28].

In addition, advancements are also required in targeted delivery systems to address skeletal involvement, improved access to affordable therapies, and the establishment of a comprehensive multidisciplinary management approach that integrates cardiopulmonary, neurological, skeletal, and psychosocial care.

Ultimately, an individualized, patient‐centered treatment approach guided by a thorough risk–benefit analysis of pharmacological and interventional options is essential for improving quality of life and long‐term outcomes in patients with Morquio syndrome.

## Author Contributions

**Pratisma Wagle:** writing – review and editing. **Manish Sah:** supervision, writing – review and editing. **Sweekar Dahal:** conceptualization, data curation, writing – review and editing. **Ramesh Raj Acharya:** supervision, writing – review and editing. **Bijay Bastola:** conceptualization, data curation, writing – original draft, writing – review and editing.

## Ethics Statement

Ethical approval was not required for the study involving humans by the local legislation and institutional requirements. Written informed consent was obtained from the patients for the publication of any potentially identifiable images or data included in this article.

## Conflicts of Interest

The authors declare no conflicts of interest.

## Data Availability

The raw data supporting the conclusions of this article will be made available by the authors without undue reservation.
